# Supplementary material for: Patterns of Intron Gain and Loss in Fungi
Source: PLoS Biol. 2004 Nov 30;2(12):e422. doi: 10.1371/journal.pbio.0020422 (PMC532390; doi:10.1371/journal.pbio.0020422)
Supplement: Table S1 — Also available at http://genes.mit.edu/NielsenEtAl/. (4.3 MB ZIP). [file pbio.0020422.st001.zip › NielsenEtAl/html/529.html]

AN2997.1.NCU03876.1.MG01013.1.FG10268.1


```
 CLUSTAL W (1.82) Multiple Sequence Alignments - Introns Inserted


Sequence 1: NCU03876.1	346 aa
Sequence 2: MG01013.1	341 aa
Sequence 3: FG10268.1	337 aa
Sequence 4: AN2997.1	336 aa
Alignment Length: 347 aa
Number Identitical Residues: 212 aa
Alignment Score (without introns) 10132


MG01013.1 	MRPILLSGH~ERALTQVK2FNPDGDLIFSVAKDQHICVWFAHNGERLGTYHGHQGAIWTV
NCU03876.1	MRPILLSGH~ERALTQIK2YNRDGDLLFSVSKDQQICVWFAHNGERLGTYHGHQGAIWTI
FG10268.1 	MRPILLAGH~ERALTQIK2FNKDGDLIFSVAKDQQICAWFSHNGERLGTYHGHVGAIWTV
AN2997.1  	MRPILLAGH0ERSLNQIK~FNRDGDLLFSVAKDKIVCAWWSANGERLGTYSGHLGAIWTV
          	******:** **:*.*:* :* ****:***:**: :*.*:: ******** ** *****:

MG01013.1 	DCDPTSTIIATGSADNTIKLWEIKTGRCLHTWEFPTAVKRVEFSPDGTRLLGVTEKRMGH
NCU03876.1	DVDPTSTILASGSADNTIRLWEIKTGRLLKTWDFPTAVKRVEFSEDGSKLLGVTEKRMGH
FG10268.1 	DVNPTSTMIASGSADNTIRLWEVKSGRLIKTWEFPTAVKRVEFNEDGTKLLGVTEKRMGY
AN2997.1  	DVSPNTVLLATGSADNSVRLWNVKTGECVKVWEFPTAVKRVEFSPDGSKVLAVTEKRMGF
          	* .*.:.::*:*****:::**::*:*. ::.*:**********. **:::*.*******.

MG01013.1 	LGTIVVLDVKLDVDAE-QNPERAMTIVCDESKATVAGWSYLAKYIIAGHEDGSVSQYDAK
NCU03876.1	LGTIVVLDIKLDVDAE-QSDEKAMTIVCEDSKATVAGWSYLSKYIIAGHEDGSVSQFDGK
FG10268.1 	LSNIVVIDINPDLNAE-QNDEKSLTIVCDESKATVAGWSALSKYIIAGHEDGSVSRYDAK
AN2997.1  	LGTIAVLDINYDGDFTNQAEEPSLRITCTESKATVAGWSYLGKYIIAGHEDGSVSQYDAK
          	*..*.*:*:: * :  .*  * :: *.* :********* *.*************::*.*

MG01013.1 	0NGDQLHSVQIHDMGSEIRDLQWSQDRTYFITASKDKTAK~LVTARDLEVLKTYPADTPL
NCU03876.1	~NGDLLYNIPIHELNQPITDLQWSHDRTYFITASKDKTSK0LITAKDLEVLKTYPADTPL
FG10268.1 	~TGDLLDNVPVHELNQPIVDLQWSPDRTYFITACKDKTSK0LISARDLEVLKTYVADTPL
AN2997.1  	0TGEQLENVQAHEFDHQINDLQFSPDRTHFLTASKDKSAK0LMSSRNLAILKTYVADTPL
          	 .*: * .:  *::.  * ***:* ***:*:**.***::* *:::::* :**** *****

MG01013.1 	NSAVITPKKDYVILGGGQAAMDVTTTSARQGKFEARFYHKIFEDEIGRVRGHFGPL0NYV
NCU03876.1	NSATITRKKDFVILGGGQAAMDVTTTSARQGKFEARFYHKIFESEIGRVRGHFGPL~NTV
FG10268.1 	NSATITPKKDFVILGGGQAAMDVTRTSARQGKFEARFYHKIFEDEIGRVRGHFGPL~NTV
AN2997.1  	NSAAITPKKEYVILGGGQAAMDVTTTSARQGKFEARFYHKVFEDEIGRVKGHFGPL~NTI
          	***.** **::************* ***************:**.*****:****** * :

MG01013.1 	AAEPNGKGYASGGEDGYVRVHQFDKGYFDFMYEVERERKNKMEQAQQ-----
NCU03876.1	AADPTGKSYASGGEDGYVRIHHFDKGYFDFMYEVERERQNKLNQQQQQTISA
FG10268.1 	AADPTGKSYASGGEDGYVRVHHFDKGYFDFNYEVERERINRMQ---------
AN2997.1  	HVHPAGTAYASGGEDGYVRVHHFDKPYFDFMYEVEREQLRR-----------
          	 ..* *..***********:*:*** **** ******: .:
```
